# N4BP3 Regulates RIG-I-Like Receptor Antiviral Signaling Positively by Targeting Mitochondrial Antiviral Signaling Protein

**DOI:** 10.3389/fmicb.2021.770600

**Published:** 2021-11-22

**Authors:** Chen Wang, Ting Ling, Ni Zhong, Liang-Guo Xu

**Affiliations:** College of Life Science, Jiangxi Normal University, Nanchang, China

**Keywords:** N4BP3, MAVS, innate immunity, RLR antiviral signaling, TRAF2

## Abstract

Mitochondrial antiviral signaling protein (MAVS), an adaptor protein, is activated by RIG-I, which is critical for an effective innate immune response to infection by various RNA viruses. Viral infection causes the RIG-I-like receptor (RLR) to recognize pathogen-derived dsRNA and then becomes activated to promote prion-like aggregation and activation of MAVS. Subsequently, through the recruitment of TRAF proteins, MAVS activates two signaling pathways mediated by TBK1-IRF3 and IKK- NF-κb, respectively, and turns on type I interferon and proinflammatory cytokines. This study discovered that NEDD4 binding protein 3 (N4BP3) is a positive regulator of the RLR signaling pathway by targeting MAVS. Overexpression of N4BP3 promoted virus-induced activation of the interferon-β (IFN-β) promoter and interferon-stimulated response element (ISRE). Further experiments showed that knockdown or knockout N4BP3 impaired RIG-I-like receptor (RLR)-mediated innate immune response, induction of downstream antiviral genes, and cellular antiviral responses. We also detected that N4BP3 could accelerate the interaction between MAVS and TRAF2. Related experiments revealed that N4BP3 could facilitate the ubiquitination modification of MAVS. These findings suggest that N4BP3 is a critical component of the RIG-I-like receptor (RLR)-mediated innate immune response by targeting MAVS, which also provided insight into the mechanisms of innate antiviral responses.

## Introduction

As the first barrier system of the body, innate immunity plays a vital role in removing foreign pathogens and guiding the body to produce effective adaptive immune responses. During the viral invasion, pathogen-associated molecular patterns (PAMPs) are initially recognized, which ultimately induces downstream effector genes, including type I interferons (IFNs) and proinflammatory cytokines (Akira et al., 2006). PAMP can be recognized by pattern recognition receptors (PRRs) to activate downstream signaling pathways. Typical pattern recognition receptors include endodermal location transmembrane Toll-like receptors (TLRs), RIG-I-like receptors (RLRs), and NOD-like receptors (NLRs) (Janeway and Medzhitov, 2002; Takeuchi and Akira, 2010). TLRs recognize single-stranded or double-stranded RNA viruses in immune cells, while RLRs recognize viruses in non-immune cells (Kawai and Akira, 2011). The RLRs family consists of three members: retinoic acid-inducible gene I (RIG-I), melanoma differentiation-associated gene-5 (MDA5), and laboratory of genetics and physiology 2 (LGP2), which recognize double-stranded RNA viruses (Kang et al., 2002; Yoneyama et al., 2004; Saito et al., 2007). All RLRs have a C-terminal domain (CTD) and an intermediate RNA helicase domain that catalyzes ATP hydrolysis (Satoh et al., 2010). RIG-I and MDA5 also have CARDs at the N-terminal that interact directly with the MAVS to activate downstream signals (Venkataraman et al., 2007; Schlee et al., 2009; Schmidt et al., 2009). The expression of RLRs is ubiquitous. Although the expression of RLRs is always low in most types of cells, once it is infected by virus or stimulated by interferon, the expression of RLR will be rapidly induced.

Mitochondrial antiviral signaling proteins (MAVS), also known as Virus-induced signaling adapter (VISA), Cardif, and IPS-1, contain a C-terminal transmembrane domain inserted into the outer membrane of mitochondria and N-terminal CARD domain (Kawai et al., 2005; Meylan et al., 2005; Seth et al., 2005; Xu et al., 2005). Activation of MAVS requires the formation of MAVS aggregates, mediated by the CARD in the cytosolic N terminus of MAVS that interacts with the tandem CARDs of RIG-I oligomers (Hou et al., 2011; Cai et al., 2014; Hui et al., 2014). Once MAVS is activated, these aggregates further recruit and activate other MAVS, thus forming large aggregates. When MAVS form prion protein aggregates, they can recruit E3 ubiquitin ligase and downstream effector proteins TNF receptor-associated factors 2 (TRAF 2), TRAF 3, and TRAF 6 to become an active “signaling body” (Liu et al., 2013). NEMO, a regulatory subunit of IKK and TBK1, is a ubiquitin sensor recruited into the MAVS/TRAFs complex through its ubiquitin-binding domain (Ea et al., 2006; Wu et al., 2006). NEMO then recruited IKK and TBK1 into the MAVS complex to activate IRF 3, IRF7, and NF-κB. The activation of IRF3 and IRF7 leads to autophosphorylation, which enters the nucleus to induce the type I IFN genes (Belgnaoui et al., 2011; Jacobs and Coyne, 2013; Liu et al., 2015; Shi et al., 2015; Vazquez et al., 2015). Similarly, activation of NF-κB phosphorylates P-65 into the nucleus and activates IFN genes expression.

As a member of the Fezzi family, the NEDD4 binding protein (N4BP3) has been reported to modulate axon and dendrite branches. It is expressed in the neural tissues of early Xenopus embryos, including the eyes, brain, and neural crest cells (Kiem et al., 2017). In addition, N4BP3 has certainly affected cell apoptosis and proliferation. N4BP3 is the interacting protein of NEDD4 ubiquitin ligase (Murillas et al., 2002). As an E3 ubiquitin ligase, NEDD4 plays a crucial role in various cellular processes through ubiquitination-mediated degradation of multiple substrates (Wang et al., 2020). However, the roles of N4BP3 in RLR-mediated signaling pathways have not been previously studied. In this study, we identified N4BP3 as an important regulator of RLR-mediated signaling pathways. Knockdown or knockout of N4BP3 can inhibit RLR-mediated activation of IRF3, induction of downstream antiviral genes, and cellular antiviral responses. Our findings reveal a previously unknown function of N4BP3 and provide insight into the mechanisms of innate antiviral responses.

## Materials and Methods

### Antibodies and Reagents

During this experiment, we used some essential antibodies: mouse monoclonal anti-Flag (Sigma-Aldrich, F3165), anti-HA antibodies (Sigma-Aldrich, H3663), anti-myc (Santa Cruz Biotechnology, sc-40), anti-IRF3 (Santa Cruz Biotechnology, sc-33641), rabbit monoclonal anti-N4BP3 (Proteintech, 16733-1-AP), anti-P65 and anti-P-P65 (Cell Signaling, #9936), Peroxidase-conjugated AffiniPure Goat anti-Rabbit IgG (H + L) (Jackson ImmunoResearch, 111-035-003), Goat anti-Mouse lgG(H + L)-HRP Conjugate (Bio-Rad, 172-1011). The dual-luciferase reporter assay system was obtained from Promega (Promega, E2610). ELISA kits were purchased from PBL Biomedical Laboratories (41415-1). Sendai virus (SeV) was kindly provided by Dr. Hong-Bing Shu (Wuhan University, Wuhan, China). Caspase inhibitor Z-VAD-FMK was purchased from Beyotime (C1202-0.02ml).

### Cell Culture, Transfection, and Viral Infection

293T and MCF7 cells were retained in Dulbecco’s modified version Eagle’s medium (DMEM; Gibco), containing 10% fetal bovine serum (FBS; Gibco) and penicillin (100 U/ml)-streptomycin (0.1 mg/ml) (Grnview) at 37°C with 5% CO_2_. Transferred the plasmids needed for the experiment into the cells by the calcium phosphate transfection method. Added SeV to the cells 12 h after plasmids transfection. When the infection time was reached, the cells were collected and lysed.

### Plasmids

Mammalian expression plasmids for human HA- or Flag-tagged RIGI, MAVS, TRAF2, TRAF3, Actb, Ubiquitin, and its mutants K48 and K63 Ubiquitin, myc-crmA, IFN-β promoter-luciferase reporter plasmid, and ISRE luciferase reporter construct were all provided by Prof. Hong-Bing Shu. The mammalian expression plasmid PRK5′-Flag or myc-tagged N4BP3 was constructed through strict molecular cloning technology. Similarly, we also got the N4BP3 plasmid of the PCMV vector. The following sequence is the primer sequence used to amplify human N4BP3 cDNA: AAAGTCGACCATGGCCACAGCCCCAGGCCCT (forward); AAAGCGGCCGCTCAGATCTTGGAGGACTCGAG (reverse).

To obtain the RNAi sequence, we can clone the corresponding double-stranded oligonucleotide target sequence into pSuper-Retro (Oligoengine, VEC-PRT-0002). The sequences were as follows:

#1: 5′-GCCAGAAGACAGCAGAGAT-3′;
#2: 5′-CCAGAAGACAGCAGAGATT-3′;
#3: 5′-GGCTTCCTATCCATGCAAA-3′;
#4: 5′-GCAAGAGCACCAAGAACAC-3′.

### CRISPR-Cas9

CRISPR-Cas9 gene-editing technology is a technology for specific DNA modification of targeted genes, and it is also a cutting-edge method used in gene editing (Sanjana et al., 2014; Shalem et al., 2014). Through this technology, we need to insert the double-stranded oligonucleotide corresponding to the target sequence into the lentiCRISPR-V2 vector to obtain the KO plasmid, which was co-transfected with packaging plasmids into 293T cells. Two days after transfection, the viruses were harvested and used to infect 293T or MCF7 cells. The infected cells were selected with puromycin (1 μg/ml) for at least 5 days. The following sequences were targeted for human N4BP3 gDNA:

#1: 5′-GGCATTGCCATGGGCAGCGT-3′;
#2: 5′-CCGGAAGGGCTTGGGCCAGC-3′.

### Co-immunoprecipitation and Immunoblotting and Native PAGE

Co-immunoprecipitation assays and western blot were performed as previously described (He et al., 2018). 293T cells were seeded in 60-mm dishes or 6-well plates and transfected with appropriate expression or Blank control plasmids. At 20 h post-transfection, cells were collected and lysed with lysis buffer containing [20 mM Tris (pH 7.5), 150 mM NaCl, 1% Triton, 1 mM EDTA, 10 μg/ml aprotinin, 10 μg/ml leupeptin, and 1 mM PMSF]. The Native PAGE was previously described (He et al., 2018).

### Dual-Luciferase Reporter Assay

The specific methods were as described in the previous experiment (He et al., 2018). 293T cells were seeded in 24-well plates and transfected on the following day by a standard calcium phosphate precipitation method. To normalize transfection efficiency, pRL-TK (Renilla luciferase) reporter plasmid was added to each transfection mixture, together with the indicated plasmids or empty vector as a control, to ensure that each well contained the same amount of total DNA. Luciferase assays were performed using a dual-specific luciferase assay kit (Promega). All reporter assays were repeated at least three times.

### ELISA

Culture supernatants of cells (∼2.5 × 10^5^) seeded on 24-well plates or serum was collected and analyzed for cytokine levels with ELISA. ELISA kits for IFN-β were purchased from PBL Biomedical Laboratories. ELISA was performed according to the manufacturer’s instructions.

### Quantitative Real-Time PCR

Quantitative Real-time PCR (Quantitative Real-time PCR) is a method that uses fluorescent chemicals to measure the total amount of product after each polymerase chain reaction (PCR) cycle in a DNA amplification reaction. Used the kit to extract total RNA from cells and performed reverse transcription to obtain cDNA. As mentioned before, quantitative real-time PCR analysis was performed (Ling et al., 2018). The primer sequences used for quantitative real-time PCR were as follows:

β*-actin* forward: GTCGTCGACAACGGCTCCGGCATG;
β*-actin* reverse: ATTGTAGAAGGTGTGGTGCCAGAT;
*IFNB1* forward: CTAACTGCAACCTTTCGAAGC;
*IFNB1* reverse: GGAAAGAGCTGTAGTGGAGAAG;
*ISG56* forward: TCATCAGGTCAAGGATAGTC;
*ISG56* reverse: CCACACTGTATTTGGTGTCTAGG;
*CXCL10* forward: GGTGAGAAGAGATGTCTGAATCC;
*CXCL10* reverse: GTCCATCCTTGGAAGCACTGCA.

### Statistical Analysis

All histogram data analysis is performed using GraphPad Prism (version 8.0; GraphPad Software, Inc., La Jolla, CA, United States), and the values are the average and variance of three independent experiments. The One-way ANOVA and Two-Way ANVOA statistical methods were used. Asterisks are considered statistically significant, ^∗^*P* < 0.05; ^∗∗^*P* < 0.01; ns means no statistical significance.

## Results

### N4BP3 Is a Positive Regulator of the RIG-I-MAVS Antiviral Signal Pathway

**FIGURE 1:**
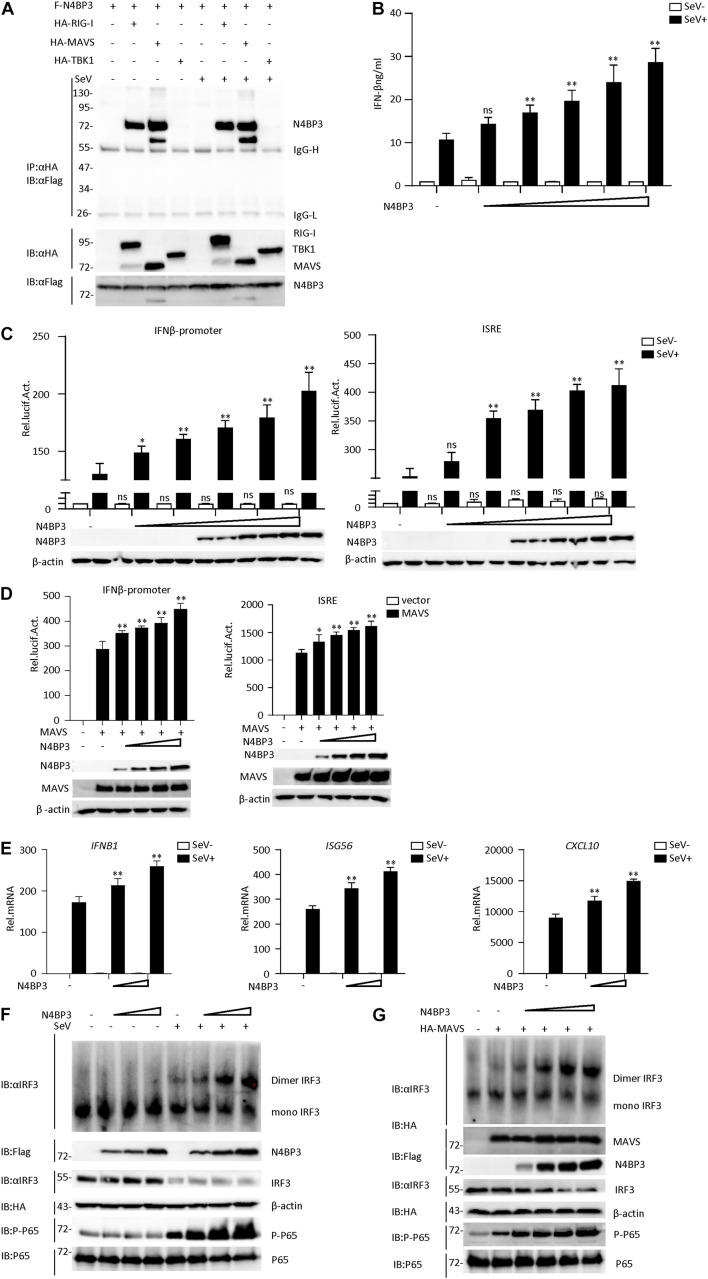
N4BP3 is a positive regulator of the RIG-I-MAVS antiviral signal pathway. **(A)** N4BP3 interacts with RIG-I and MAVS. 293T were transfected with the indicated plasmid (3 μg each). 24 h after transfection, cells were left uninfected or infected with SeV before harvested and lysed to perform Co-immunoprecipitation assays with anti-HA, following immunoblotting assay with anti-Flag. Expression levels of the proteins were analyzed by immunoblot analysis of the lysates with anti-HA and anti-Flag antibodies. **(B)** N4BP3 promotes the production of IFN-β. Transfect different doses (0, 0.01, 0.1, 0.5, 1.0, and 1.5 μg) of N4BP3 expression plasmid into 24-well plates. After 12 h of transfection, the cells were left uninfected or infected with SeV for 12 h, and the concentration of the cytokines in the culture supernatant was measured by ELISA. The data table shown were presented as mean ± SD, *n* = 3, *P < 0.05, **P < 0.01. **(C)** N4BP3 promotes the activation of the IFN-β promoter and ISRE induced by SeV. The IFN-β promoter or ISRE luciferase plasmid (0.05 μg) was transfected into 293T cells (∼1 × 10^5^) cultured in a 24-well plate, and different doses (0, 0.01, 0.1, 0.5, 1.0, and 1.5 μg each) of N4BP3 expression plasmids were transfected simultaneously. After 12 h of transfection, cells were left uninfected or infected with SeV for 12 h, and the dual-luciferase reporter gene experiment was performed. The data table shown were presented as mean ± SD, *n* = 3, *P < 0.05, **P < 0.01. **(D)** Overexpression of N4BP31 enhances MAVS-mediated activation of IFN-β promoter and ISRE. Before the reporter gene detection, MAVS (0.5 μg) and different doses of N4BP3 (0, 0.01, 0.1, 1.0, and 1.5 μg each) and reporter gene plasmids (0.05 μg each) were transfected into 293T cells for 20 h. The data table shown were presented as mean ± SD, *n* = 3, *P < 0.05, **P < 0.01. **(E)** Under SeV induction, N4BP3 enhances the expression of IFNB1, ISG56, and CXCL10 at the mRNA level. 293T cells were transfected with different doses (0, 0.5, and 1.0 μg each) of the indicated plasmid. After 12 h of transfection, the cells were left uninfected or infected with SeV for 12 h and then subjected to qRT-PCR detection, while the actin was used for normalization. The data table shown were presented as mean ± SD, *n* = 3, *P < 0.05, **P < 0.01. **(F)** N4BP3 promotes SeV-induced IRF3 dimer formation. 293T cells were transfected with N4BP3 plasmids (0.5, 1.0, and 2.0 μg each). 12 h after transfection, the cells were left uninfected or infected with SeV for 12 h. IRF3 dimers or monomers were analyzed by Native PAGE for western blot analysis. **(G)** N4BP3 promotes MAVS-mediated IRF3 dimer formation and phosphorylation of P65. 293T cells were transfected with different doses (0, 0.5, 1.0, 1.5, and 2.0 μg each) of N4BP3 and MAVS (1.5 μg each) plasmids for 20 h. Western blot analysis is like panel **(F)**.

The RLR signaling pathway plays an indispensable role in the process of virus infection. To further explore the RIG-I-MAVS antiviral signal mechanism, the yeast two-hybrid screening was performed by using the full length of MAVS as the bait, a few novel genes, including N4BP3, were identified as MAVS associated partners. To further confirm whether N4BP3 interacted with MAVS, the Flag-N4BP3 expression plasmid was co-transfected with empty vector, HA-labeled RIG-I, MAVS, and TBK1 plasmids, respectively, in 293T human kidney cells. The co-immunoprecipitation results indicated that N4BP3 interacted with RIG-1 and MAVS but not with TBK1 (Figure 1A and Supplementary Figure 1A).

By conducting a dual-luciferase reporter and ELISA assay in 293T cells, we found that N4BP3 could potentiate SeV-induced activation of IFN-β promoter and ISRE in a dose-dependent manner (Figures 1B,C). Consistently, N4BP3 overexpression dose-dependently increased MAVS-mediated activation of IFN-β promoter and ISRE (Figure 1D). The real-time fluorescence quantitative PCR experiments indicated overexpression of N4BP3 potentiated SeV-induced transcription of downstream genes, including IFNB1, ISG56, CXCL10 (Figure 1E). Overexpression of N4BP3 further increased SeV-induced and MAVS-mediated IRF3 dimerization and P-65 phosphorylation (Figures 1F,G). These results suggest that N4BP3 potentiates the RNA virus-triggered induction of downstream antiviral genes.

### Knockdown of N4BP3 Inhibits RIG-I-MAVS Antiviral Signaling Pathway

**FIGURE 2:**
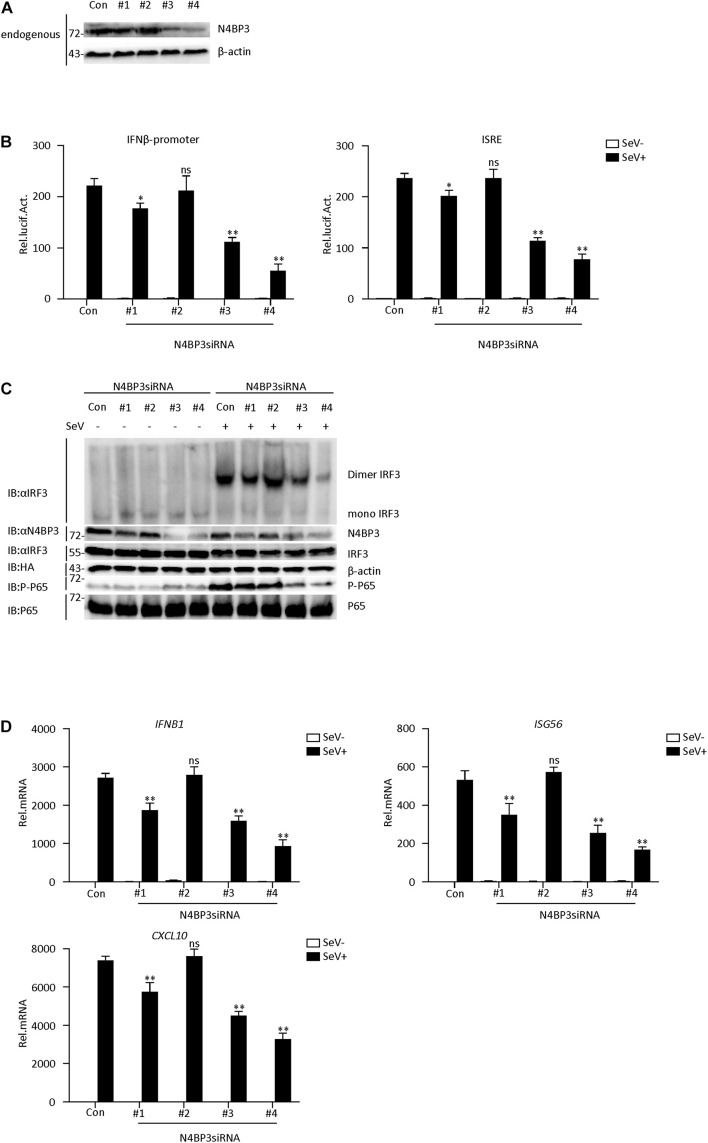
Knockdown of N4BP3 inhibits RIG-I-MAVS antiviral signaling pathway. **(A)** The effect of N4BP3 shRNA plasmid on the expression of endogenous N4BP3. Co-transfected into 293T cells with control or N4BP3 shRNA plasmid and HA-Actb. Western blot analysis was performed with corresponding antibodies. **(B)** Knockdown of N4BP3 inhibits SeV-induced activation of IFN-β promoter and ISRE. 293T cells were transfected with the indicated N4BP3 shRNA (1.5 μg) and reporter gene plasmids (0.05 μg). After 12 h of transfection, the cells were left uninfected or infected with SeV for 12 h before luciferase assays were performed. The data table shown were presented as mean ± SD, *n* = 3, *P < 0.05, **P < 0.01. **(C)** Knockdown of N4BP3 inhibits SeV-induced IRF3 dimer formation and phosphorylation of P65. 293T cells were transfected with control siRNA plasmid or designated N4BP3 siRNA plasmid (4.0 μg each). 12 h after transfection, the cells were left uninfected or infected with SeV for 12 h. IRF3 dimers or monomers were analyzed by Native PAGE for western blot analysis. **(D)** Under SeV induction, knocking down N4BP3 blocked the expression of IFNB1, ISG56, and CXCL10 at the mRNA level. Transfected 293T cells with the indicated shRNA plasmid. After 12 h of transfection, the cells were left uninfected or infected with SeV for 12 h and then subjected to qRT-PCR detection. The data table shown were presented as mean ± SD, *n* = 3, *P < 0.05, **P < 0.01.

To further determine its physiological role in the RIG-I-MAVS antiviral signaling mechanism, we used RNA interference techniques to reduce endogenous N4BP3 expression levels. We constructed four N4BP3-RNAi plasmids (#1, #2, #3, and #4), which could knock down the expression of N4BP3 to various degrees. Transient transfection and endogenous expression experiments indicated that shN4BP3 #1, #3, #4 efficiently downregulated N4BP3 expression at the protein level as suggested by immunoblot analysis (Figure 2A). In reporter assays, knockdown of N4BP3 significantly inhibited SeV-triggered activation of the IFN-β promoter and ISRE (Figure 2B). Native PAGE Western blotting showed that the reduction of endogenous N4BP3 expression could significantly reduce the formation of SeV-induced IRF3 dimerization and the phosphorylation of P65, which is a hallmark of IRF3 activation (Figure 2C). The degree of inhibition was related to how well shRNA reduced the expression of endogenous N4BP3. Consistently, knockdown of N4BP3 markedly inhibited SeV-induced transcription of IFNB1, ISG56, CXCL10 genes (Figure 2D). These data suggest that N4BP3 regulates virus-induced signaling mediated by RIG-I. From the above experimental data, #4 had the highest efficiency in reducing the expression of endogenous N4BP3, and the effect of inhibiting the RIG-I-MAVS signaling pathway was the best. Therefore, the subsequent experiment chose #4 to proceed.

### Knockout of N4BP3 Inhibits RIG-I-MAVS Antiviral Signaling Pathway

**FIGURE 3:**
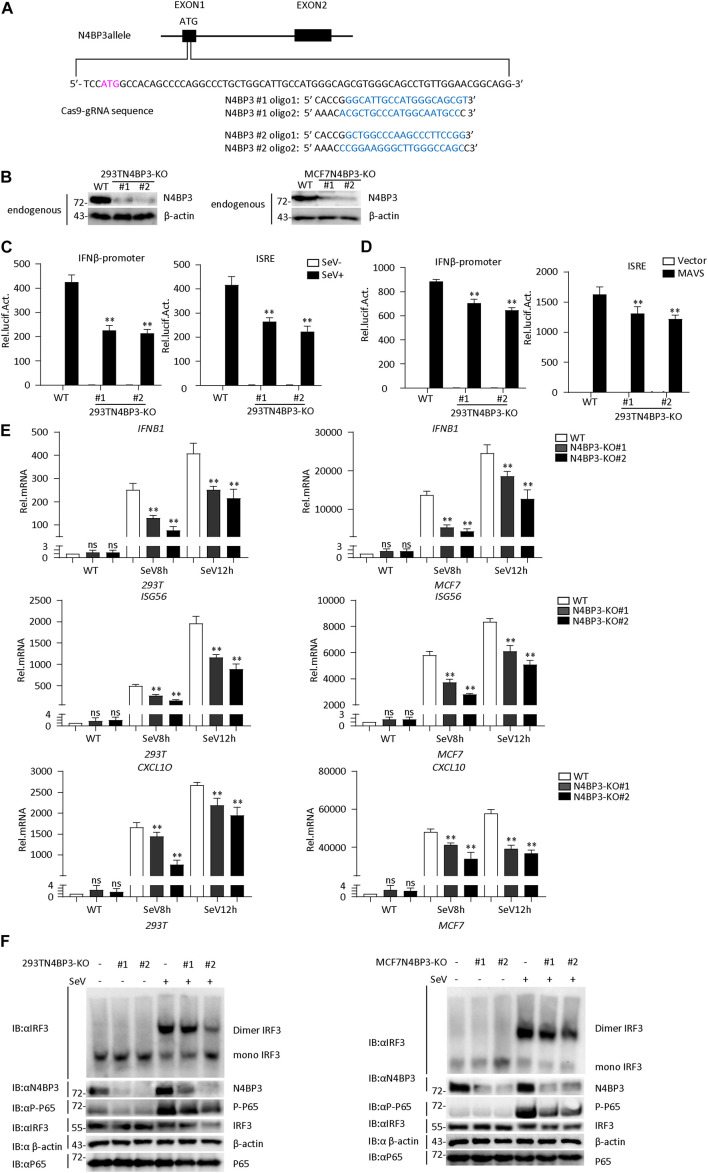
Knockout of N4BP3 inhibits RIG-I-MAVS antiviral signaling pathway. **(A)** A scheme for CRISPR/Cas9-mediated genome editing of the N4BP3 gene locus. **(B)** Knockout efficiencies of N4BP3. Immunoblotting was used to verify the expression of N4BP3 protein in N4BP3-deficient 293T cells and MCF7 cells and control cells. **(C)** Knockout of N4BP3 in 293T cells can inhibit SeV-induced activation of IFN-β promoter and ISRE. 293T WT and two different N4BP3KO 293T cells were transfected with IFN-β promoter or ISRE luciferase plasmid (0.05 μg), as well as pRL-TK (Renilla luciferase plasmid; 0.05 μg). After 12 h of transfection, the cells were left uninfected or infected with SeV for 12 h and then subjected to luciferase detection. The data table shown were presented as mean ± SD, *n* = 3, ^∗^*P* < 0.05, ^∗∗^*P* < 0.01. **(D)** Knockout of N4BP3 in 293T cells can inhibit MAVS-mediated activation of IFN-β promoter and ISRE. 293T WT and two different N4BP3KO 293T cells were transfected with IFN-β promoter or ISRE luciferase plasmid (0.05 μg) and pRL-TK (Renilla luciferase plasmid; 0.05 μg) and empty vector or MAVS expression vector. After 20 h of transfection, then subjected to luciferase detection. The data table shown were presented as mean ± SD, *n* = 3, ^∗^*P* < 0.05, ^∗∗^*P* < 0.01. **(E)** Under SeV induction, knocking out N4BP3 blocked the expression of IFNB1, ISG56, and CXCL10 at the mRNA level. Control cells and N4BP3-deficient 293T cells and MCF7 cells were left uninfected or infected with SeV for 12 h and then subjected to qRT-PCR detection, while β-actin was used as an internal control. The data table shown were presented as mean ± SD, *n* = 3, ^∗^*P* < 0.05, ^∗∗^*P* < 0.01. **(F)** Knockout of N4BP3 inhibits SeV-induced IRF3 dimer formation and phosphorylation of P65. Control cells and N4BP3-deficient 293T cells and MCF7 cells were left uninfected or infected with SeV for 12 h. IRF3 dimers or monomers were analyzed by Native PAGE for western blot analysis.

To confirm the role of N4BP3 in RLR-mediated signaling, we generated two different mixed N4BP3-deficient 293T and MCF7 cells lines (KO#1 and KO#2) by CRISPR-Cas9. The knockout effect in 293T and MCF7 cells can be confirmed at DNA and protein levels (Figures 3A,B and Supplementary Figure 1C).

In reporter assays, the SeV-induced and MAVS-mediated activation of IFN-β promoter and ISRE reduced in two mix N4BP3-deficient cells compared with their control cells (Figures 3C,D). Consistently, knockout of N4BP3 significantly inhibited SeV-induced transcription of downstream genes such as IFNB1, ISG56, CXCL10 (Figure 3E). In addition, the level of virus-induced IRF3 dimerization and P65 phosphorylation in N4BP3-deficient 293T and MCF7 cells was significantly lower than that in the control group (Figure 3F). The above experiments further demonstrate that N4BP3 positively regulates the RIG-I-MAVS antiviral signal pathway.

### N4BP3 Promotes the Polyubiquitination of MAVS

**FIGURE 4:**
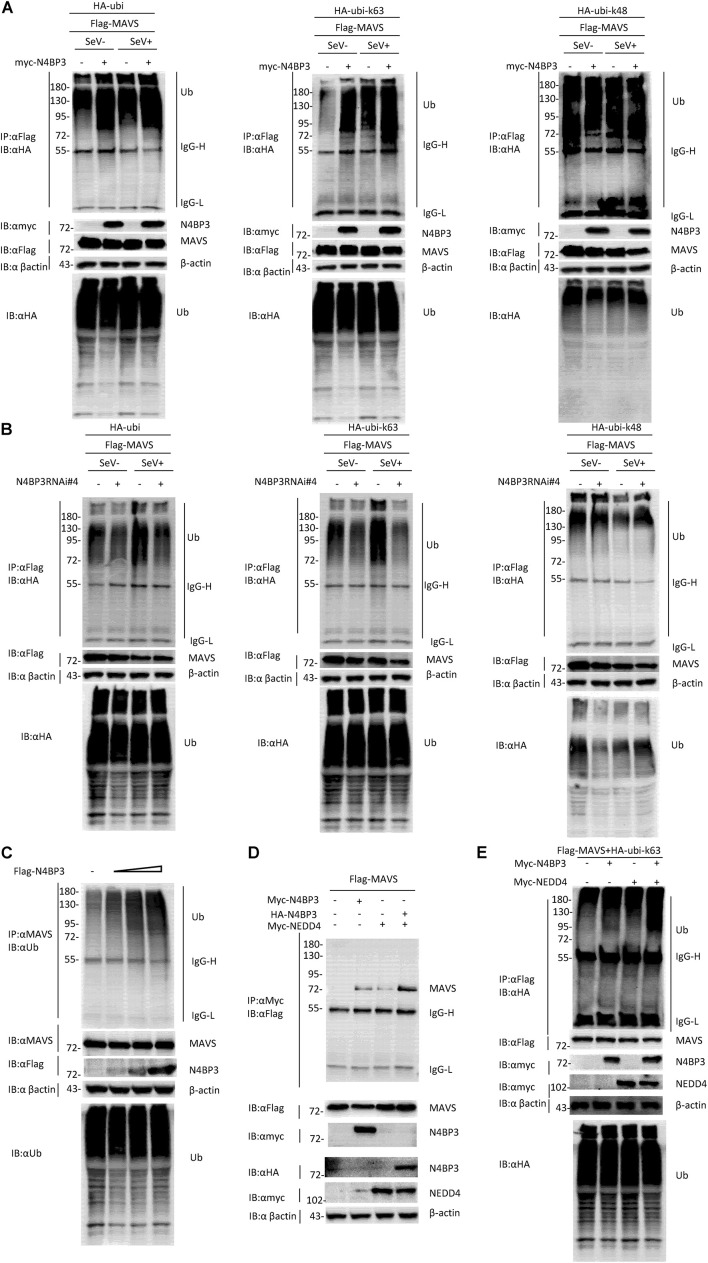
N4BP3 promotes the polyubiquitination of MAVS. **(A)** N4BP3 promotes the polyubiquitination of MAVS. 293T cells were transfected with the indicated plasmids. After 12 h of transfection, the cells were stimulated with or without SeV for 12 h, and the cells were collected and lysed. The indicated antibodies were used for co-immunoprecipitation and immunoblotting analysis. Most lysates are used for anti-Flag beads immunoprecipitation and western blotting analysis with anti-HA antibodies. A small portion of the lysate is used for routine western blotting to check the expression of each plasmid. **(B)** N4BP3 knockdown prevents MAVS polyubiquitination. The methods of co-immunoprecipitation and immunoblotting were like panel **(A)**. **(C)** N4BP3 promotes endogenous MAVS ubiquitination. 293T transfected with the indicated plasmids. 24 h after transfection, cell lysates were immunoprecipitated with anti-MAVS. The immunoprecipitates were analyzed by immunoblots with anti-ubiquitin, anti-MAVS, or anti-Flag as indicated. **(D)** NEDD4 enhances the interaction between MAVS and N4BP3. 293T transfected with Flag-tagged MAVS, in the presence or absence of Myc-tagged N4BP3, Myc-tagged NEDD4, and HA-tagged N4BP3 for 24 h, followed by co-immunoprecipitation and immunoblotting analysis with the indicated antibodies. **(E)** NEDD4 promotes the ubiquitination of MAVS by N4BP3. The N4BP3, NEDD4 plasmids, and other plasmids were transfected into 293T cells, and after 20 h, the cells were collected and lysed. The methods of co-immunoprecipitation and immunoblotting were like panel **(A)**.

Ubiquitination is a key regulatory mechanism of the virus-induced innate immune response. To further investigate the mechanism by which N4BP3 positively regulates RIG-I-MAVS antiviral signal transduction, we conducted experiments on the effect of N4BP3 on the polyubiquitination of MAVS under the SeV induction. We co-transfected 293T cells with N4BP3 and MAVS, together with HA-tagged Ubi, and its mutant k48/k63, with only one lysine at positions 48 and 63, respectively, and observed that N4BP3 overexpression stimulated MAVS linked linear ubiquitination (Figure 4A). Furthermore, endogenous co-immunoprecipitation experiments indicated that N4BP3 promoted the polyubiquitination of MAVS (Figure 4C). In contrast, N4BP3 deficiency markedly decreased MAVS-mediated total polyubiquitination and k63-linked polyubiquitination (Figure 4B).

Our experiment results show that the partner of ubiquitin ligase Nedd4, Nedd4-binding protein 3 (N4BP3), interacts with MAVS and functions as a positive regulator to promote K63 polyubiquitination of MAVS in the RLR signaling pathway. These results point to the possibility that NEDD4 is a ubiquitin ligase that links N4BP3 to MAVS in RLR antiviral signaling. To test this, we examined whether the NEDD4-N4BP3 complex interacts with MAVS. The transient transfected co-immunoprecipitation experiments results showed that the NEDD4-N4BP3 complex, compared to NEDD4 alone or N4BP3 alone, showed more intense interaction with MAVS (Figure 4D). These results suggest that N4BP3 might recruit NEDD4 to MAVS in the RLR antiviral signaling. Then, we explore whether NEDD4 affects MAVS ubiquitination. The co-immunoprecipitation experiments results showed that N4BP3 or NEDD4 alone could promote K63-linked MAVS ubiquitination, and the NEDD4-N4BP3 complex could have more capability to enhance the k63 ubiquitination of MAVS (Figure 4E). All these results suggest that the possibility that N4BP3 recruits the NEDD4 ubiquitin ligase to MAVS to enhance its antiviral signaling by augmenting K63 polyubiquitination of MAVS in the RLR signaling pathway.

### N4BP3 Strengthens the Interaction Between MAVS and TRAF2

**FIGURE 5:**
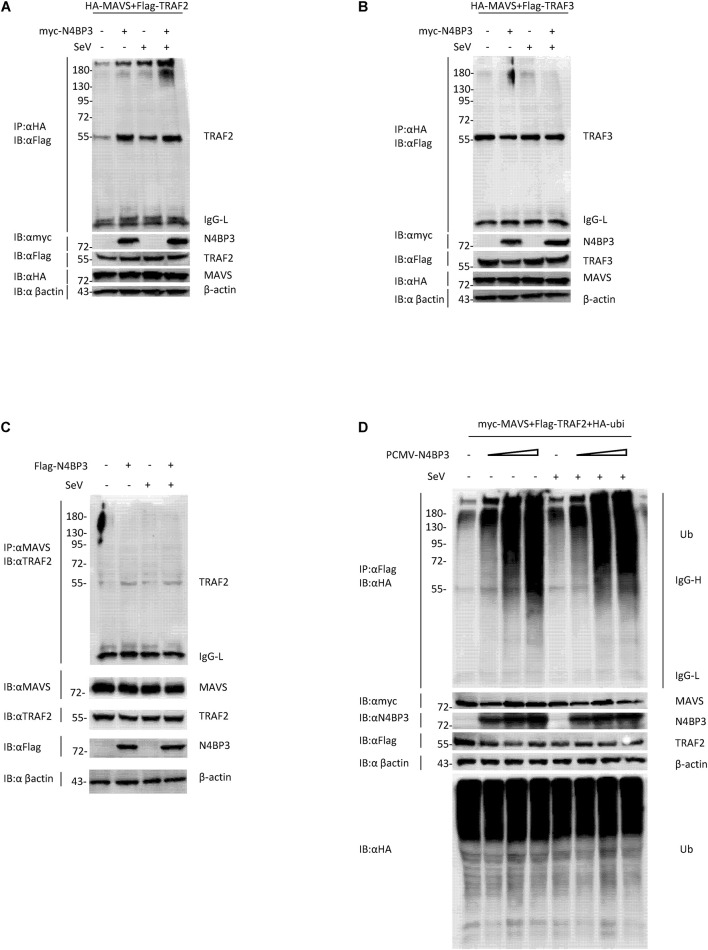
N4BP3 strengthens the interaction between MAVS and TRAF2. **(A)** N4BP3 can promote the interaction between MAVS and TRAF2. Co-transfected HA-labeled MAVS, Flag-labeled TRAF2, and, respectively, transfected myc-labeled N4BP3 into 293T cells. After 12 h of transfection, the cells were left uninfected or infected with SeV for 12 h, and the cells were collected and lysed. The indicated antibodies were used for co-immunoprecipitation and immunoblotting analysis. Most lysates are used for anti-HA beads immunoprecipitation and western blotting analysis with anti-Flag antibodies. A small portion of the lysate is used for routine western blotting to check the expression of each plasmid. **(B)** N4BP3 does not affect the MAVS-TRAF3 complex. Co-immunoprecipitation and immunoblot analyses were similarly performed as in panel **(A)**. **(C)** N4BP3 promotes the interaction between endogenous MAVS and TRAF2. 293T transfected with the indicated plasmids. Coimmunoprecipitation and immunoblots were performed with the indicated antibodies. **(D)** N4BP3 can promote the polyubiquitination of TRAF2, a downstream complex of MAVS, and the effect of polyubiquitination is dose-dependent on N4BP3. 293T transfected with the indicated plasmids, followed by co-immunoprecipitation and immunoblotting analysis with the indicated antibodies.

After the virus infects cells, cytoplasmic RIG-I senses viral RNA promotes MAVS aggregation and recruits tumor necrosis factor receptor-associated factor (TRAF) family proteins, including TRAF2 and TRAF3 and TRAF6, activation of the downstream signaling pathway to stimulate antiviral immune responses (Hou et al., 2011; Liu et al., 2013; Hui et al., 2014). When we co-transfected 293T cells with HA-tagged MAVS and Flag-tagged TRAF2 or TRAF3, in the presence or absence of myc-tagged N4BP3, we found that N4BP3 overexpression attenuated the extent of MAVS co-precipitation with Flag-TRAF2, suggesting that N4BP3 strengthens this interaction (Figures 5A,B). The endogenous experiment was consistent with the above results (Figure 5C).

Previous research reports pointed out that the ubiquitination of TRAF2 is unintentionally important in the activation process of NF-κB, and the activation of NF-κB induces the production of downstream antiviral factors (Gudi et al., 2009; Li et al., 2009). We co-transfected myc-labeled MAVS, Flag-labeled TRAF2, HA-labeled ubiquitin plasmids and, respectively, transfected different doses of PCMV-N4BP3 into 293T cells for co-immunoprecipitation experiments. The results insinuate that N4BP3 can promote the polyubiquitination of TRAF2, a downstream complex of MAVS, and the effect of polyubiquitination was dose-dependent on N4BP3 (Figure 5D). In summary, these findings indicate that N4BP3 can promote the stability of the MAVS and TRAF2 complex and enhance the polyubiquitination of TRAF2 to achieve the positive regulation of the RIG-I-MAVS antiviral signal.

## Discussion

The RIG-I-MAVS signal pathway is an indispensable antiviral innate immune signal transduction, which requires further investigation. As the central hub of the RLR signaling pathway, MAVS plays a role in linking up and down. In-depth research on MAVS will provide useful value to the RLR signaling pathway.

This study found that overexpression of N4BP3 potentiated the SeV-induced activation of ISRE and the IFNβ promoter, whereas knockdown and knockout of N4BP3 inhibited the SeV-induced transcription of downstream antiviral genes.

The N-terminal CARD domain of MAVS mediates its interaction with RLR and the important downstream targets, including TNF receptor-associated factor 2 (TRAF2). The results show that N4BP3 can indeed promote the interaction between MAVS and TRAF2. TRAF2 contains the N-terminal ring finger domain and the C-terminal highly conserved domain. Its polyubiquitination exerts biological effects and activates the NF-κB mechanism, ultimately leading to the production of IFN-β (Vallabhapurapu and Karin, 2009; Yao et al., 2014). In this study, we found that N4BP3 can enhance the polyubiquitination of TRAF2 and thus positively regulate RIG-I-MAVS signal transduction. However, how N4BP3 strengthens the interaction between MAVS and TRAF needs to be further studied.

The NEDD4 ubiquitin ligase family mainly includes NEDD4, NEDD4L, ITCH, SMURF1, SMURF2, WWP1, WWP2, NEDL1, and NEDL2 family members (Scheffner and Kumar, 2014). Among them, ITCH regulates innate antiviral immunity through the PCBP2-ITCH axis defines MAVS degradation (You et al., 2009), E3 ligase Nedd4L positively regulates antiviral immunity by catalyzing K29-linked cysteine ubiquitination of TRAF3 (Gao et al., 2021), Smurf2 targeted MAVS for K48-linked ubiquitination for its degradation (Pan et al., 2014). Our experimental results showed that overexpression of N4BP3 increases the k63-linked MAVS polyubiquitination by recruiting the NEDD4 ubiquitin ligase to MAVS for its K63-linked polyubiquitination, suggesting that N4BP3-NEDD4 complex might play an essential role to regulate RIG-I-MAVS-mediated antiviral signaling positively. Next, we need to investigate further how NEDD4 regulates the process of antiviral innate immunity by targeting MAVS.

We also found that when MAVS and N4BP3 were co-transfected, an unexpected N4BP3 degradation protein band appeared. However, this degradation band of N4BP3 disappeared when we treated the cells with crmA or the pan-caspase inhibitor BD-fmk (Supplementary Figure 1B), suggesting that MAVS-mediated N4BP3 degradation is caspase-dependent.

In summary, our research identified that N4BP3 positively regulated the antiviral innate immune signal pathway mediated by RIG-I-MAVS and provided a novel insight into the role of N4BP3 in MAVS. Further work should determine whether N4BP3 ubiquitinates MAVS through the ubiquitin link enzyme NEDD4.

## Data Availability Statement

The original contributions presented in the study are included in the article/Supplementary Material, further inquiries can be directed to the corresponding author/s.

## Author Contributions

L-GX designed the research. CW, TL, and NZ performed the experiments. L-GX and CW did data analysis and discussion. CW and L-GX wrote the manuscript. All authors contributed to the article and approved the submitted version.

## Conflict of Interest

The authors declare that the research was conducted in the absence of any commercial or financial relationships that could be construed as a potential conflict of interest.

## Publisher’s Note

All claims expressed in this article are solely those of the authors and do not necessarily represent those of their affiliated organizations, or those of the publisher, the editors and the reviewers. Any product that may be evaluated in this article, or claim that may be made by its manufacturer, is not guaranteed or endorsed by the publisher.

## Supplementary Material

Supplementary Figure 1 **(A)** N4BP3 interacts with RIG-I and MAVS. 293T cells were transfected with the indicated plasmids before co-immunoprecipitation and immunoblotting analysis. **(B)** MAVS and N4BP3 induce fragmentation, and this is Caspase-dependent. 293T cells were transfected with the indicated plasmids for 24 h before immunoblotting analysis was performed. **(C)** Genomic DNA was extracted from the 293T cells and MCF7 cells of the N4BP3 KO #1 clone and KO #2 clone, and then the DNA fragments around the target area were amplified by polymerase chain reaction (PCR) and sequenced.
