# Supplementary material for: N4BP3 Regulates RIG-I-Like Receptor Antiviral Signaling Positively by Targeting Mitochondrial Antiviral Signaling Protein
Source: Front Microbiol. 2021 Nov 22;12:770600. doi: 10.3389/fmicb.2021.770600 (PMC8646042; doi:10.3389/fmicb.2021.770600)
Supplement: Supplementary Figure 1 — (A) N4BP3 interacts with RIG-I and MAVS. 293T cells were transfected with the indicated plasmids before co-immunoprecipitation and immunoblotting analysis. (B) MAVS and N4BP3 induce fragmentation, and this is Caspase-dependent. 293T cells were transfected with the indicated plasmids for 24 h before immunoblotting analysis was performed. (C) Genomic DNA was extracted from the 293T cells and MCF7 cells of the N4BP3 KO #1 clone and KO #2 clone, and then the DNA fragments around the target area were amplified by polymerase chain reaction (PCR) and sequenced. [file Presentation_1.pptx]

## Slide 1
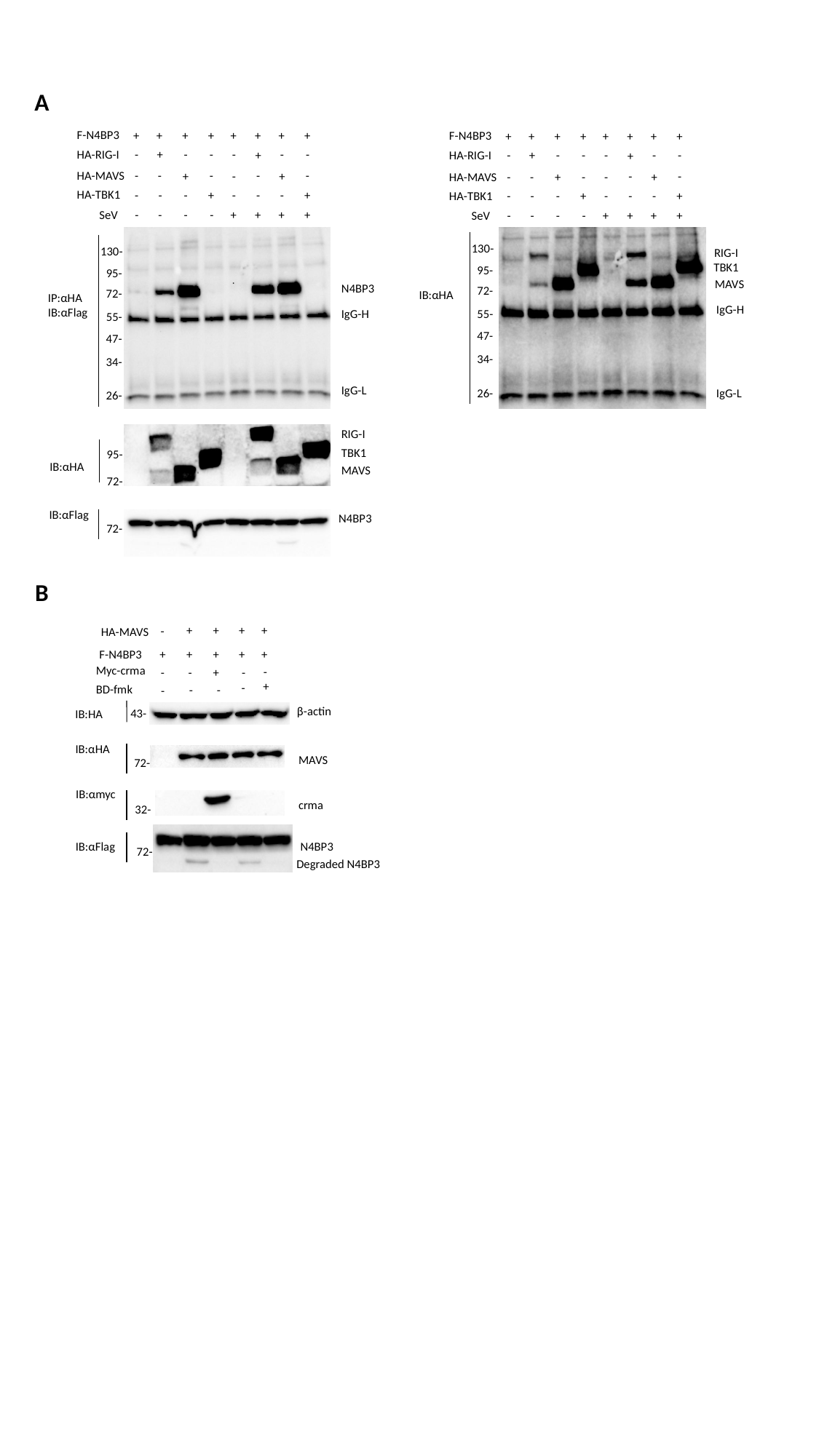

A
F-N4BP3
+
-
-
-
-
+
+
-
-
-
+
+
+
-
-
-
+
+
+
-
-
+
+
+
F-N4BP3
+
-
-
-
-
+
+
-
-
-
+
+
+
-
-
-
+
+
+
-
-
+
+
+
-
HA-RIG-I
-
-
-
-
HA-RIG-I
-
-
-
-
-
HA-MAVS
+
+
-
-
HA-MAVS
+
+
HA-TBK1
-
-
+
+
HA-TBK1
-
-
+
+
+
+
-
-
SeV
+
+
-
-
SeV
130-
130-
RIG-I
TBK1
95-
95-
MAVS
N4BP3
72-
72-
IB:αHA
IP:αHA
IB:αFlag
IgG-H
55-
IgG-H
55-
47-
47-
34-
34-
IgG-L
26-
IgG-L
26-
RIG-I
TBK1
95-
IB:αHA
MAVS
72-
IB:αFlag
N4BP3
72-
B
+
+
+
+
-
HA-MAVS
+
+
+
F-N4BP3
+
+
Myc-crma
-
+
-
-
-
+
-
BD-fmk
-
-
-
β-actin
43-
IB:HA
IB:αHA
MAVS
72-
IB:αmyc
crma
32-
IB:αFlag
N4BP3
72-
Degraded N4BP3

## Slide 2
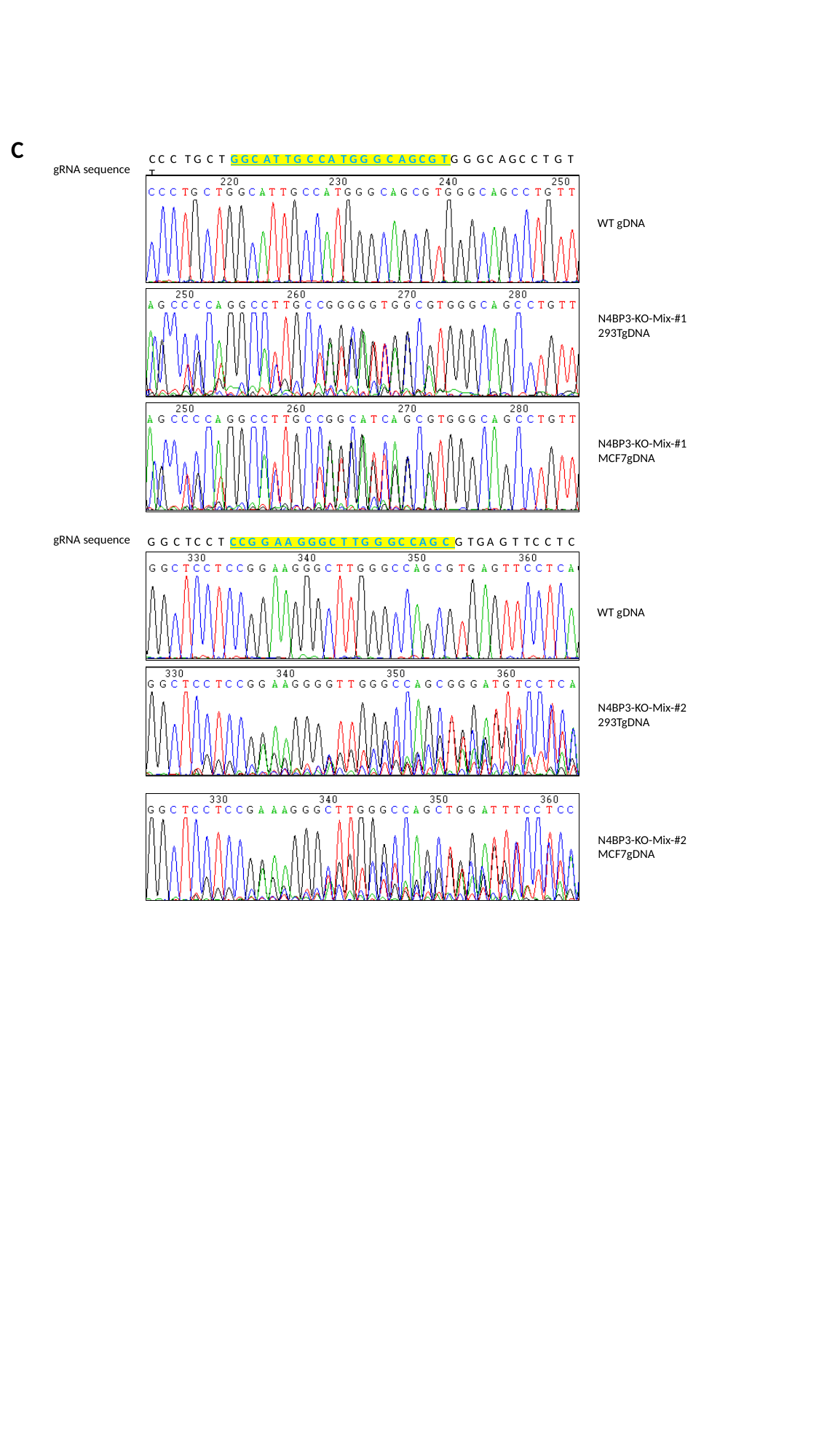

C
C C C T G C T G G C A T T G C C A T G G G C A G C G T G G G C A G C C T G T T
gRNA sequence
WT gDNA
N4BP3-KO-Mix-#1
293TgDNA
N4BP3-KO-Mix-#1
MCF7gDNA
gRNA sequence
G G C T C C T C C G G A A G G G C T T G G G C C A G C G T G A G T T C C T C A
WT gDNA
N4BP3-KO-Mix-#2
293TgDNA
N4BP3-KO-Mix-#2
MCF7gDNA
